# Fish Karyome version 2.1: a chromosome database of fishes and other aquatic organisms

**DOI:** 10.1093/database/baw012

**Published:** 2016-03-15

**Authors:** Naresh Sahebrao Nagpure, Ajey Kumar Pathak, Rameshwar Pati, Iliyas Rashid, Jyoti Sharma, Shri Prakash Singh, Mahender Singh, Uttam Kumar Sarkar, Basdeo Kushwaha, Ravindra Kumar, S. Murali

**Affiliations:** 1 Division of Molecular Biology and Biotechnology; 2 Division of Fish Taxonomy and Resources, National Bureau of Fish Genetic Resources, Canal Ring Road, PO—Dilkusha, Lucknow 226002, UP, India

## Abstract

A voluminous information is available on karyological studies of fishes; however, limited efforts were made for compilation and curation of the available karyological data in a digital form. ‘Fish Karyome’ database was the preliminary attempt to compile and digitize the available karyological information on finfishes belonging to the Indian subcontinent. But the database had limitations since it covered data only on Indian finfishes with limited search options. Perceiving the feedbacks from the users and its utility in fish cytogenetic studies, the Fish Karyome database was upgraded by applying Linux, Apache, MySQL and PHP (pre hypertext processor) (LAMP) technologies. In the present version, the scope of the system was increased by compiling and curating the available chromosomal information over the globe on fishes and other aquatic organisms, such as echinoderms, molluscs and arthropods, especially of aquaculture importance. Thus, Fish Karyome version 2.1 presently covers 866 chromosomal records for 726 species supported with 253 published articles and the information is being updated regularly. The database provides information on chromosome number and morphology, sex chromosomes, chromosome banding, molecular cytogenetic markers, etc. supported by fish and karyotype images through interactive tools. It also enables the users to browse and view chromosomal information based on habitat, family, conservation status and chromosome number. The system also displays chromosome number in model organisms, protocol for chromosome preparation and allied techniques and glossary of cytogenetic terms. A data submission facility has also been provided through data submission panel. The database can serve as a unique and useful resource for cytogenetic characterization, sex determination, chromosomal mapping, cytotaxonomy, karyo-evolution and systematics of fishes.

**Database URL:** http://mail.nbfgr.res.in/Fish_Karyome

## Introduction

A huge amount of karyological information have been generated worldwide on fishes and other aquatic organisms of aquaculture importance. Some efforts were made earlier to compile and document the scattered information in the form of books, such as ‘Database on Fish Chromosome’ (1) ‘Fish Chromosome Atlas’ (2) and ‘Fish Karyotypes’ (3). The exponential growth of fish karyological data during the last two decades pressed the need to manage the data by developing digital databases. Nagpure *et al.* (4) described the development of the first web-based database named as ‘Fish Karyome: A Karyological Information Network on Indian fishes’ by digitizing the ‘Fish Chromosome Atlas’. This database was developed by applying SQL Server 2000, Microsoft’s ASP.NET-2008 and Macromedia’s FLASH technologies under Windows 7 operating platform. It had limitations since it covered data only on Indian finfish with limited search options, lack of information on the conservation status and the absence of data submission panel.

Observing the growing interest and usefulness of the system and feedbacks from different researchers, efforts were made to upgrade the existing Fish Karyome into a more user friendly system covering the available cytogenetic information on fishes and other aquatic organisms, so that system may provide its visibility at the global scale. The new version was developed by using platform-independent technologies Linux, Apache, MySQL and PHP (pre hypertext processor) (LAMP) and provides information on chromosome number and morphology, karyotype formula, sex chromosomes, and cytogenetic markers such as nucleolar organizer regions, constitutive heterochromatic bands, etc. supported by fish and karyotype images. Additionally, it holds information on taxonomy, geographical distribution, conservation status, protocol for chromosome preparation and banding, fluorescence *in situ* hybridization (FISH) technique, chromosome number of model organisms and glossary of cytogenetic terms. Search options such as keyword search and search based on habitat, family, conservation status and chromosome number widen as well as narrow down the scope to search and view the information of interest. At present, the database of Fish Karyome version 2.1 contains 866 chromosomal records of 726 fish and other animals of aquaculture importance representing 146 families and 47 orders. The present article discusses about the newer version and changes brought out in the wider perspective.

## Materials and methods

### Data collection and curation

In total, 253 published literature from diverse sources that included peer reviewed articles, published books, data resources and Ph.D. works were surveyed and chromosomal information on fishes including few aquatic animals were collected and curated manually (Table 1). Thus, information on chromosome number and morphology, sex chromosomes, karyotype formula and cytogenetic markers such as nucleolar organizer regions and constitutive heterochromatic bands along with images of karyotypes and species were compiled.

**Table 1.** List of sources used for data collection and curation

S. no.	Information type	Main resources	Supported resources
1	ChromosomaI information	PubMed http://www.ncbi.nlm.nih.gov/pubmed	Different publishing articles on cytogenetics, chromosome, Ichthyology and cell biology, etc.
		Peer-reviewed articles	The Scientific Electronic Library Online—SciELO: http://www.scielo.br/
		Published Books	Perspectives in Cytology and Genetics https://books.google.co.in/books/
		Ph.D. thesis	
2	General information	FishBase www.fishbase.org/ WoRMS http://www.marinespecies.org/	USGS Science for a changing World http://nas.er.usgs.gov/queries/
			The Global Invasive Species Database http://www.issg.org
			Encyclopedia of Life: http://eol.org/pages/
			Animal Diversity Web: http://animaldiversity.org
			WorldFish http://www.worldfishcenter.org/databases
3	Synonyms	FishBase www.fishbase.org/	The Global Invasive Species Database http://www.issg.org
4	Importance	FishBase www.fishbase.org/	International Game Fish Association https://www.igfa.org/Fish/Fish-Database.aspx
5	Conservation status	The IUCN Red List of threatened species http://www.iucnredlist.org/	Wildscreen Arkive http://www.arkive.org
6	Taxonomy	NCBI	Food and Agriculture Organization of the United Nations for a world without hunger http://www.fao.org
			Interagency Taxonomic Information System (ITIS): http://www.itis.gov/
			WoRMS: www.marinespecies.org/
			FishBase www.fishbase.org/
7	Genome size information		Animal Genome Size Database (AGSD) www.genomesize.com

In order to avoid copyright and other ethical issues for including images and chromosomal information into the database, permission has been obtained from the publishers/authors wherever necessary. All compiled information was documented on the digital data sheets and was used as data source for developing the database. The other information of species such as general, ecology, taxonomy and conservation status were collected from authentic data sources such as FishBase (5) and WoRM (6), NCBI (7) and IUCN Red List Status (8) and verified using the supported resources as described in Table 1. This information compiled with karyological information using a modified Perl program InformationParser.pl (9) was applied in preparing datasets, as per schema and populating data into the respective tables of the database.

### Version number

The version of the system increases to 1 by changing major part, the application of different technology and developing environment, and the addition of new modules or features. The alteration into the designing of the database and data expansion by inclusion of new information and other improvement activity in the schema force to increase version by 0. 1. In this way, the previous system was upgraded and version enhanced from 1 to 2. Similarly, the database structure and data expansion enhanced the version 2 by point 1. Hence, finally the present version of Fish Karyome was represented by version 2.1.

## Design and development

### Database

**Figure 1.**
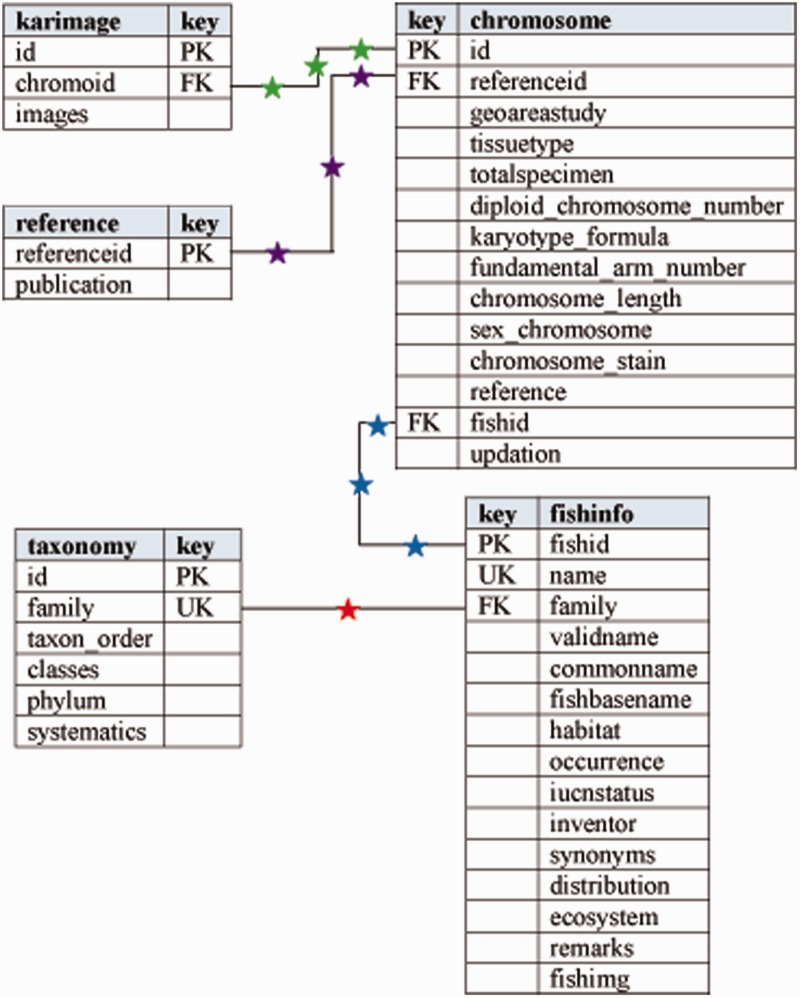
Entity relationship diagram of Fish Karyome database.

For developing the new version of Fish Karyome, the database of an earlier version was migrated into MySQL relational database management system and thereafter it was restructured by creating more tables covering the different attribute types. Relationships between the tables were modified using unique, primary and foreign keys. Figure 1 shows the entity relationship (E-R) model of the database. The table ‘fishinfo’, covers information on taxon, biogeography and conservation status and table ‘taxonomy’ covers the systematic information on the species. Similarly, table ‘chromosome’ holds karyological information of fishes and table ‘karimage’ covers the karyotype image records in the database. Finally, table ‘reference’ collects the references of records.

### Web interface

In order to facilitate the user to work with the database, a user-friendly interactive web interface was designed and developed using PHP, JavaScripts, CSS (cascading style sheets) and HTML technologies.

### Utility tools for information retrieval

Different utility tools facilitating search, browsing and retrieving the information of interest from the database were designed and included in the web interface. Two types of search options were included to retrieve and view the information. These are keyword search and search through control interfaces. The keyword search option includes as ‘Search’ accepts keywords such as ‘fish name’, ‘common name’, ‘family’, ‘habitat’, ‘synonyms’ and ‘discoverer’ of species as input and retrieves information from the database accordingly. The later was facilitated through ‘Browse’ option included in the web interface to view the information of interest by selecting either of the choices likes ‘species’, ‘family’, ‘chromosome number’, ‘conservation status’, ‘habitat’ and ‘model fishes’.

### Data submission panel, data management and feedback

In order to submit data into the database, the menu item ‘Submission’ enables creation of new user and allows the registered user to login in the system. The system administrator provides authenticity to newly signed up user to be recognized as a registered user of Fish Karyome for login into the system for data submission. The user submitted data have been screened and tested by the experts in their relevant fields before inclusion into the main database. This feature of the database increases accuracy and authenticity of the volume of data being submitted from different parts of the world. Further the feedback submission panel was also designed and included in the web interface of the system to receive the feedback about the system along with personal information of the user in a digital form.

## Results and discussion

A total of 866 chromosomal information of 726 species of fishes and other aquatic animal were compiled and curated manually by collecting information from 253 published articles. All the curated information populated into the database developed using MySQL relational database management system presently covers karyological information of fishes (678) and other aquatic organisms of economic importance, such as echinoderms (6) molluscs (21) and arthropods (21). Besides, karyological information of threatened species (63) including the extinct ones were also covered. The system through its web interface presents different utility tools that provide the ability to work with the database. The home page of the web interface also includes options to retrieve and view the information on 24 model organisms, protocols for chromosome preparation and FISH, glossary of important cytogenetic terms and the feedback option for users to voluntarily submit comments about the system. Under data management option, Fish Karyome version 2.1 provides not only data management but also data submission facility only by the authorized users. The data submitted by the user get inserted into the main database after approval by the database administrator. This option can be invoked through user login window included into the web interface of the system.

### Data expansion

The previous version of Fish Karyome covering karyological information was restricted only to the Indian finfishes. The continuously growing interest and constant feedback from the scientific community towards Fish Karyome impugned for the expansion of information by upgrading the complete system. Thus, in the present version, volume of chromosomal records was increased from 377 to 866 chromosomal records covering 726 species whilst in earlier version species coverage was 171. Similarly, the volume of supported references was increased from 32 to 253. In this way, the numbers of taxonomic order and family of covered species were got updated. The number of orders was increased from 10 to 47 and family from 43 to 146 and this information can be viewed by clicking on the numeric values of current status on the home page. Besides, these efforts, there are still scattered karyological information in the form of written publications on fishes and other important aquatic animal reported from different part of the world that shows the utility of this system for reconciliation of information in future years and volunteer submission of data by the researchers.

### Updating and inserting new records

The database is frequently updated by addition of new records and updating the existing records. Mining, compiling and curating information on fish karyology from scientific publications is a tedious task and takes longer time for preparation of the data. Therefore, in order to expedite the growth of the database, Fish Karyome facilitates data submission by the registered users for submitting the published records on chromosomal information.

### Keyword search

**Figure 2.**
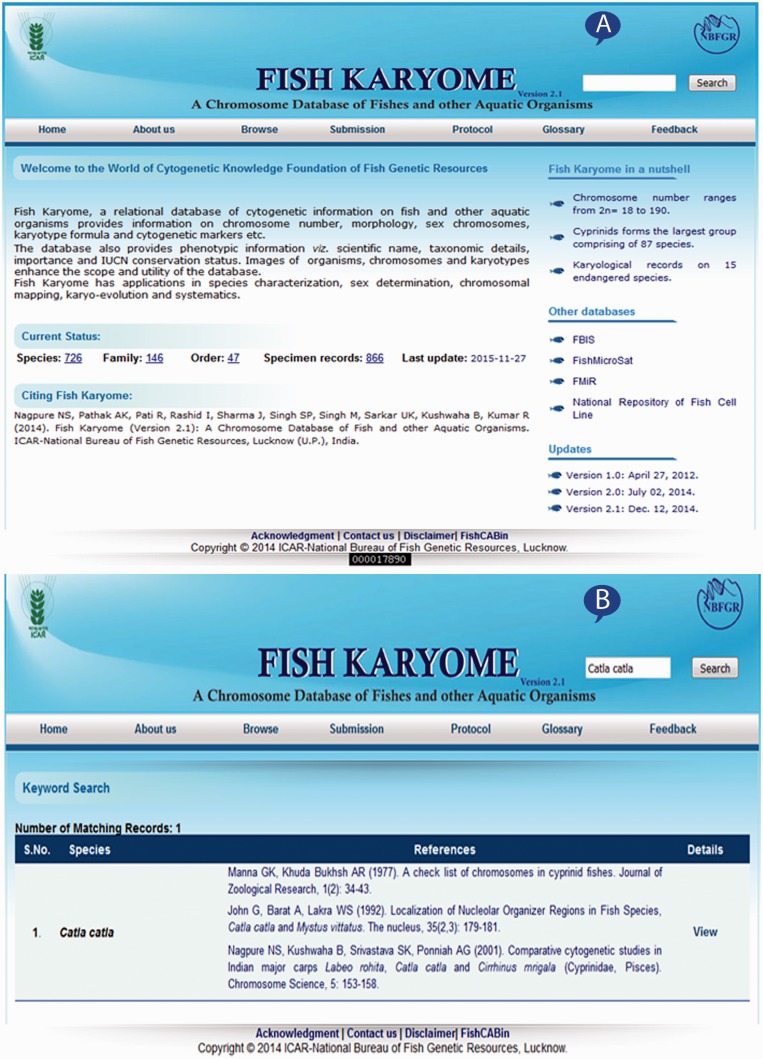
Web interface of Fish Karyome: (**A**) home page and (**B**) key word search.

The home page of Fish Karyome version 2.1 includes keyword search and other options that allow the user to work with the system as presented in Figure 2A. The keyword search allows retrieving records from the database matching the keyword typed by the user. It accepts keywords such as value of either of the species name, common name, family name, habitat and conservation status for retrieving information from the database. The search result displays information from the database and also presents the option to view the detail information, e.g. if the user types the ‘*Catla* *catla*’ keyword in the search box and performs the click event on the ‘Search’ button, it displays the records matching only this word (Figure 2B).

### Browsing information on aquatic organisms

**Figure 3.**
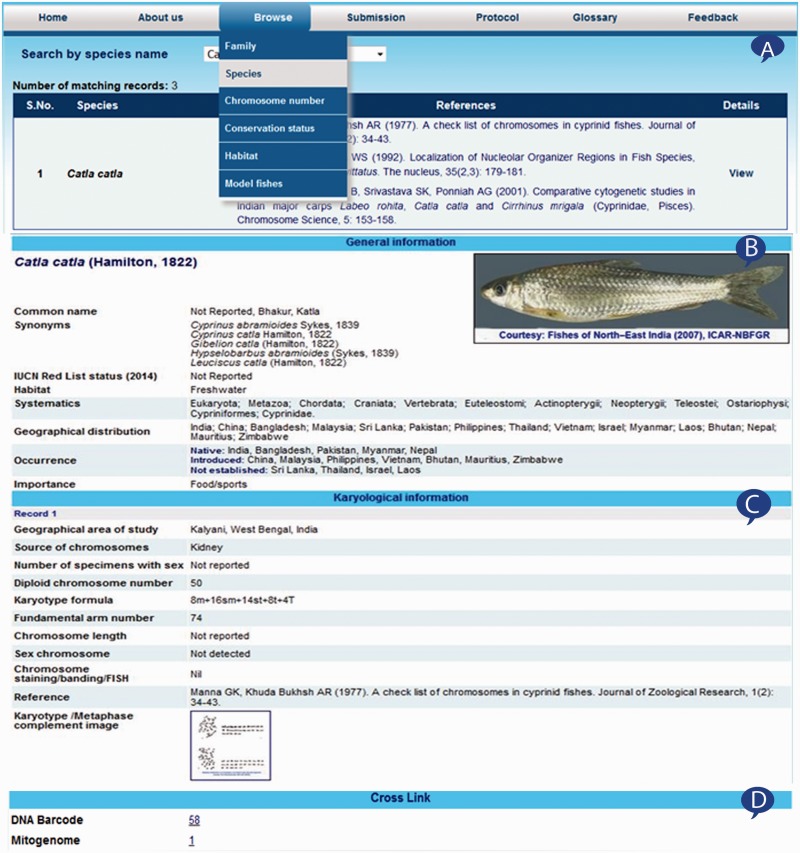
Browsed information from Fish Karyome: (**A**) information summarized into a table, (**B**) species information, (**C**) karyological information and (**D**) linked with other data resources.

The ‘Browse’ option included in the web interface of the system provides another choice to view the information from the database. This option provides the ability to search and view records from the database using either of the choices such as species name, family name, chromosome number, conservation status, habitat and model fishes. The selection of choice displays species name and reference of its karyological in a table (Figure 3A). The ‘View’ hyper link under the details column of summary table presents general and karyological information in separate section. The general information provides details on species name, discoverer, valid name, synonyms, common name, habitat, conservation status, occurrence, distribution and systematics along with fish image (Figure 3B). The karyological information part as presented in Figure 3C comprised information presented on geographical area of study, source of chromosomes, number of specimens with sex, diploid chromosome number, karyotype formula, fundamental arm number, chromosome length, sex chromosome, chromosome staining/banding/FISH, reference(s) and karyotype images. Both types of information displayed in the windows allow the user to generate the report of the displayed information, which can be saved or printed for later use. Moreover, DNA barcode and mitogenome information for species linked with respective public databases FBIS (10) and FMiR (11) are presented in Figure 3D.

### Viewing information on model organisms

Zebrafish, fugu, medaka and a diversified group of fish species have been used as models for human disease and identification of genetic traits (12). A web interface of ‘Model fishes’ has been included under ‘Browse’ menu item for viewing their chromosomal information. The detail view of each model species is linked with AGSD database (13) in the cross-link section along with FBIS and FMiR links.

### Viewing information on protocols and glossary of cytogenetic words

To enhance the scope of the system and its utility even to students as a study material, the protocol option included in the web interface of the system provides the material on chromosome preparation and banding as well as FISH. Additionally, a glossary of some important cytogenetic terms were also covered which can be viewed through the ‘Glossary’ menu item included in the web interface of the system.

### Documentation of user’s feedback

Feedback from the user is one of the essential components to improve the efficacy of the system. The ‘Feedback’ option collects the feedbacks from different users on a data entry enabled digital feedback form.

## Conclusion

Fish Karyome version 2.1 is a new database driven information system for fishes and other aquatic organisms. The system presently provides the ability to view the cytogenetic information of 726 fish species belonging to 146 families of 47 orders covered into 866 records. Besides, the database of the system covers other valuable information, such as habitat, distribution, occurrence, conservation status and taxonomy of each species. The system with different interactive search options provides the ability to retrieve and view the information of interest from the database. Undoubtedly, inclusion of chromosomal information on model fishes, protocols for chromosome preparation and FISH, glossary of important cytogenetic terms enhances the scope and multifaceted utility of the system. The data submission and management panel by the authenticated users is one of the cornerstones of this system, which not only secures the databases but also keeps the updating mechanism of the database in continuation. Thus, this new version is expected to be more useful for researchers, academicians and even students working in the field of fish cytogenetic and genomics.
